# Metabolic response as assessed by ^18^F‐fluorodeoxyglucose positron emission tomography‐computed tomography does not predict outcome in patients with intermediate‐ or high‐risk rhabdomyosarcoma: A report from the Children's Oncology Group Soft Tissue Sarcoma Committee

**DOI:** 10.1002/cam4.3667

**Published:** 2020-12-19

**Authors:** Douglas J. Harrison, Yueh‐Yun Chi, Jing Tian, Pooja Hingorani, Leo Mascarenhas, Geoffrey B. McCowage, Brenda J. Weigel, Rajkumar Venkatramani, Suzanne L. Wolden, Torunn I. Yock, David A. Rodeberg, Andrea A. Hayes‐Jordan, Lisa A. Teot, Sheri L. Spunt, William H. Meyer, Douglas S. Hawkins, Barry L. Shulkin, Marguerite T. Parisi

**Affiliations:** ^1^ University of Texas MD Anderson Cancer Center Houston TX USA; ^2^ University of Florida Gainesville FL USA; ^3^ Children's Hospital Los Angeles and University of Southern California Keck School of Medicine Los Angeles CA USA; ^4^ The Children's Hospital at Westmead Weastmead NSW Australia; ^5^ University of Minnesota/Masonic Cancer Center Minneapolis MN USA; ^6^ Baylor College of Medicine/Dan L Duncan Comprehensive Cancer Center Houston TX USA; ^7^ Memorial Sloan Kettering Cancer Center New York NY USA; ^8^ Massachusetts General Hospital Cancer Center Boston MA USA; ^9^ East Carolina University Greenville NC USA; ^10^ UNC Lineberger Comprehensive Cancer Center Chapel Hill NC USA; ^11^ Boston Children's Hospital Boston MA USA; ^12^ Lucile Packard Children's Hospital Stanford University Palo Alto CA USA; ^13^ University of Oklahoma Health Sciences Center Oklahoma City OK USA; ^14^ Seattle Children's Hospital Seattle WA USA; ^15^ Saint Jude Children's Research Hospital Memphis TN USA

**Keywords:** chemotherapy, complete metabolic response, maximum standard uptake value (SUVmax), pediatric, positron emission tomography, rhabdomyosarcoma

## Abstract

**Background:**

Strategies to optimize management in rhabdomyosarcoma (RMS) include risk stratification to assign therapy aiming to minimize treatment morbidity yet improve outcomes. This analysis evaluated the relationship between complete metabolic response (CMR) as assessed by ^18^F‐fluorodeoxyglucose positron emission tomography‐computed tomography (FDG‐PET) imaging and event‐free survival (EFS) in intermediate‐risk (IR) and high‐risk (HR) RMS patients.

**Methods:**

FDG‐PET imaging characteristics, including assessment of CMR and maximum standard uptake values (SUVmax) of the primary tumor, were evaluated by central review. Institutional reports of SUVmax were used when SUVmax values could not be determined by central review. One hundred and thirty IR and 105 HR patients had FDG‐PET scans submitted for central review or had SUVmax data available from institutional report at any time point. A Cox proportional hazards regression model was used to evaluate the relationship between these parameters and EFS.

**Results:**

SUVmax at study entry did not correlate with EFS for IR (*p* = 0.32) or HR (*p* = 0.86) patients. Compared to patients who did not achieve a CMR, EFS was not superior for IR patients who achieved a CMR at weeks 4 (*p* = 0.66) or 15 (*p* = 0.46), nor for HR patients who achieved CMR at week 6 (*p* = 0.75) or 19 (*p* = 0.28). Change in SUVmax at week 4 (*p* = 0.21) or 15 (*p* = 0.91) for IR patients or at week 6 (*p* = 0.75) or 19 (*p* = 0.61) for HR patients did not correlate with EFS.

**Conclusion:**

Based on these data, FDG‐PET does not appear to predict EFS in IR or HR‐RMS. It remains to be determined whether FDG‐PET has a role in predicting survival outcomes in other RMS subpopulations.

## Graphical abstract

This analysis evaluated the relationship between complete metabolic response (CMR) as assessed by ^18^F‐fluorodeoxyglucose positron emission tomography‐computed tomography (FDG‐PET) imaging and event‐free survival (EFS) in intermediate‐risk (IR) and high‐risk (HR) RMS patients. FDG‐PET parameters including baseline SUVmax, change in SUVmax, and CMR do not predict EFS in IR or HR‐RMS.

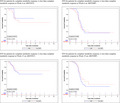

**Lay summary**

This manuscript reports the ^18^F‐fluorodeoxyglucose Positron Emission Tomography‐Computed Tomography (FDG‐PET) imaging data from two large, prospective, Children's Oncology Group Studies—ARST0531 and ARST08P1—of pediatric patients with intermediate‐ and high‐risk rhabdomyosarcoma, respectively. The study examined several FDG‐PET variables on imaging submitted from patients enrolled on each study to determine if FDG‐PET imaging could be correlated with event‐free survival (EFS) in rhabdomyosarcoma. None of the variables tested were found to correlate with EFS in rhabdomyosarcoma suggesting the imaging modality does not have a role in predicting outcome in intermediate‐ or high‐risk rhabdomyosarcoma.

## 1 INTRODUCTION

Rhabdomyosarcoma (RMS) is the most common soft tissue sarcoma (STS) in children and adolescents with approximately 350 cases documented in the United States each year.^1^ Clinical features present at diagnosis can stratify patients into low‐, intermediate‐, and high‐risk groups to predict outcome and modulate treatment intensity.^2^, ^3^ Patients with low‐risk and intermediate‐risk (IR) RMS have a relatively favorable long‐term event‐free survival (EFS) of approximately 90% and 60%, respectively, whereas patients with high‐risk (HR) disease have poor outcomes with a 5‐year EFS of approximately 30%.^4^, ^5^, ^6^ Recent studies to improve outcomes in RMS have explored novel ways of stratifying risk to identify patients that may require more intensive or novel therapies.^6^, ^7^, ^8^, ^9^, ^10^

Response to induction chemotherapy is a well‐established predictor of survival in many pediatric cancers, including acute lymphoblastic leukemia, Hodgkin lymphoma, osteosarcoma, and Ewing sarcoma.^11^, ^12^, ^13^, ^14^, ^15^, ^16^ Both radiographic and histologic response have been used to adapt treatment intensity in an overall effort to minimize toxicity for lower risk patients and escalate therapy for higher risk patients. Radiographic response to treatment (as measured by change in tumor size), however, has not proven to be a reliable predictor of outcome in RMS, limiting the ability to identify patients who could benefit from more intensive or novel therapy.^17^, ^18^, ^19^

Metabolic activity as assessed by ^18^F‐FDG‐PET/CT (FDG‐PET) has recently been shown to improve accuracy of staging in pediatric RMS.^20^, ^21^ Preliminary data from a single institution suggested that a complete metabolic response (CMR) as assessed by FDG‐PET following radiation therapy for local control may predict local relapse‐free survival (LRFS) in pediatric patients with Group III RMS.^22^ A follow‐up analysis by the same group confirmed these findings in a larger cohort of RMS patients showing that metabolic response by FDG‐PET predicted EFS, overall survival, and local tumor control.^23^ The predictive value of FDG‐PET response, however, has not been evaluated in a prospective multi‐institutional RMS clinical trial.

In this analysis, we present the FDG‐PET imaging data from two large, prospective Children's Oncology Group (COG) studies for newly diagnosed IR RMS and HR RMS: ARST0531 (Randomized Study of Vincristine, Dactinomycin, and Cyclophosphamide (VAC) versus VAC Alternating with Vincristine and Irinotecan (VI) for Patients with Intermediate‐Risk Rhabdomyosarcoma) and ARST08P1 (A Pilot Study to Evaluate Novel Agents (Temozolomide and Cixutumumab) in Combination with Intensive Multi‐Agent Interval Compressed Therapy for Patients with High‐Risk Rhabdomyosarcoma). The purpose of this analysis was to determine whether complete metabolic response as assessed by FDG‐PET imaging correlates with EFS in these two subpopulations of RMS patients.

## 2 PATIENTS AND METHODS

### 2.1 Patient population

Details of the design, eligibility criteria, treatment, and outcome for ARST0531 (NCT00354835) and ARST08P1 (NCT01055314) have been previously published.^5^, ^7^ ARST0531 enrolled newly diagnosed patients with IR RMS defined as patients with non‐metastatic (Group I‐III) alveolar RMS arising at any site (Stage 1–3) and incompletely excised (Group III) embryonal RMS arising in an unfavorable site (Stage 2–3). Patients received 42 weeks of either VAC alone or alternating cycles of VAC with VI. Only patients with measurable disease at study entry (Clinical Group III) were included in this analysis. Radiation therapy (50.4 Gy in 1.8 Gy fractions for Clinical Group III patients) was given at week 4 of therapy. Patients enrolled on both treatment arms were combined for the purpose of this FDG‐PET analysis, as there was no statistical difference in EFS between the two treatment arms.^5^ FDG‐PET imaging was optional for all patients and was recommended prior to chemotherapy, at week 4, and at week 15. ARST08P1 enrolled newly diagnosed patients with HR RMS defined as patients with metastatic (Stage 4/Group IV) alveolar or embryonal RMS. Patients were treated with a multi‐agent chemotherapy backbone which included two cycles of VI (Weeks 1–6), followed by six cycles of alternating interval compressed vincristine, doxorubicin, and cyclophosphamide (VDC) and ifosfamide and etoposide (IE) (weeks 7–19). Two VI cycles were repeated at weeks 20–25 and weeks 47–51. Interval‐compressed VDC/IE cycles were administered again at weeks 28 through 34 followed by four cycles of VAC administered every 3 weeks during weeks 35–46. Patients were enrolled in sequential pilot cohorts to receive the described chemotherapy backbone in conjunction with cixutumumab or temozolomide with the primary aim to evaluate the feasibility of the combination.^7^ Radiation therapy was administered at week 20 to the primary tumor as well as to sites of metastatic disease at the discretion of the treating institution. FDG‐PET imaging was performed prior to chemotherapy, at week 6, and week 19 (prior to radiation therapy) if clinically indicated and available at the treating institution.

### 2.2 Measurement of response and definition of endpoints

We evaluated both FDG‐PET tumor response and baseline maximum standard uptake values. Two nuclear imaging physicians (MTP and BLS) centrally reviewed FDG‐PET response. Response was classified according to European Organization for Research and Treatment of Cancer criteria.^24^ CMR was defined as complete resolution of abnormal FDG uptake within the tumor region defined on baseline scan. SUVmax values were assessed by central review or institutional report if central review was not available. SUVmax was determined by manually drawing a region of interest over the area of FDG activity corresponding to the tumor in question. Quality assurance of the treating institution included documentation of: blood glucose level before injection, injected 18F‐FDG dose, time of injection, time that image acquisition began, patient weight and height for determination of SUV, and assurance that the DICOM header was intact. Both transverse CT files and transverse ^18^F‐FDG‐PET attenuation corrected files were required.

### 2.3 Statistical analysis methods

EFS was defined as the time from study enrollment to disease progression, disease recurrence, occurrence of a second malignant neoplasm, or death from any cause. EFS for patients who did not experience disease progression or death was censored at the subject's last contact date. Follow‐up is current as of 31 December 2018. The Kaplan–Meier method was utilized to estimate the EFS.^25^ EFS was compared between groups using the log‐rank test.^26^ A Cox proportional hazards regression model was used to evaluate SUVmax and ratio of SUVmax.^27^ Software SAS 9.4^®^, was used for the analysis.

## 3 RESULTS

### 3.1 Patient demographics and disease characteristics

**FIGURE 1:**
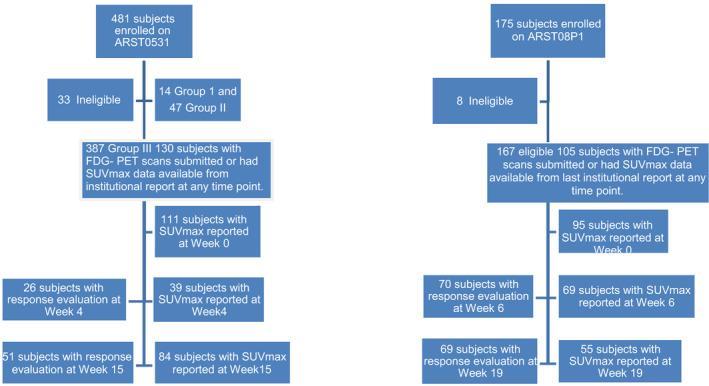
Disposition of patients enrolled on ARST0531 and ARST08P1 and the distribution of available FDG‐PET imaging data

Demographic data for the evaluated patient cohort are shown in Table 1 for ARST0531 and ARST08P1. Figure 1 shows the disposition of patients enrolled on ARST0531 and ARST08P1 and the distribution of available FDG‐PET imaging data. Of the 387 eligible patients with Clinical Group III disease enrolled on ARST0531, 130 patients had FDG‐PET scans submitted for central review or had SUVmax data available from institutional report at any time point. Twenty‐six IR patients had week 4 and 51 patients had week 15 FDG‐PET imaging available for response evaluation. FDG‐PET SUVmax at the primary tumor site was reported by the treating institution and evaluated by central imaging review if available. On the IR study, SUVmax data were analyzed for 111 patients prior to chemotherapy, 39 patients at week 4, and 84 patients at week 15. Of the 167 eligible and evaluable patients enrolled on ARST08P1, 105 patients had FDG‐PET scans submitted for central review or had SUVmax data available from institutional report at any time point. Seventy HR patients had week 6, and 69 patients had week 19 FDG‐PET imaging available for response evaluation. FDG‐PET SUVmax of the primary tumors was reported by the treating institution and evaluated by central review if available. On the HR study, SUVmax data were analyzed for 95 patients prior to chemotherapy, 69 patients at week 6, and 55 patients at week 19.

**TABLE 1:** Clinical features and treatment of patients from ARST0531 (N = 130) and ARST08P1 (N = 105) with FDG‐PET response or SUVmax data available

Clinical characteristic	Number of 0531 patients (%)	Number of 08P1 patients (%)
Gender		
Male	67 (51.5%)	53 (50.5%)
Female	63 (48.5%)	52 (49.5%)
Age, years		
0–0.99	3 (2.3%)	1 (1.0%)
1–9.99	67 (51.5%)	22 (21.0%)
≥10	60 (46.2%)	82 (78.1%)
Race		
White	99 (76.2%)	78 (74.3%)
Black	15 (11.5%)	11 (10.5%)
Asian	4 (3.1%)	3 (2.9%)
Other/unknown	12 (9.2%)	13 (12.4%)
Ethnicity		
Hispanic or Latino	17 (13.1%)	23 (21.9%)
Not Hispanic or Latino	108 (83.1%)	78 (74.3%)
Unknown	5 (3.9%)	4 (3.8%)
RMS histology		
Embryonal	67 (51.5%)	23 (21.9%)
Alveolar	57 (43.9%)	75 (71.4%)
NOS/unknown	6 (4.6%)	7 (6.7%)
Maximum tumor size, cm^a^		
<5	55 (42.6%)	21 (20.0%)
5–9.99	65 (50.4%)	49 (46.7%)
≥10	9 (7.0%)	35 (33.3%)
Regional lymph node status		
N0	100 (76.9%)	48 (45.7%)
N1	30 (23.1%)	56 (53.3%)
Not evaluated/unknown	N/A	1 (1.0%)
Primary site		
Orbit	1 (0.8%)	0 (0%)
Head or neck	4 (3.1%)	5 (4.8%)
Parameningeal	76 (58.5%)	13 (12.4%)
GU, bladder/prostate	12 (9.2%)	5 (4.8%)
GU, non‐bladder/prostate	0 (0%)	9 (8.6%)
Extremity	20 (15.4%)	30 (28.6%)
Retroperitoneal/perineal	15 (11.5%)	25 (23.8%)
Trunk	2 (1.5%)	9 (8.6%)
Other or Missing Data	0 (0%)	9 (8.6%)
Fusion status^b^		
FOXO1−	76 (58.5%)	26 (24.8%)
FOXO1+	44 (33.9%)	38 (36.2%)
Unknown	10 (7.7%)	41 (39.1%)
Treatment		
VAC	65 (50.0%)	N/A
VAC/VI	65 (50.0%)	N/A
IMC‐A12: 3 mg/kg dose	N/A	16 (15.2%)
IMC‐A12: 6 mg/kg dose	N/A	12 (11.4%)
IMC‐A12: 9 mg/kg dose	N/A	37 (35.2%)
Temozolomide	N/A	40 (38.1%)

Abbreviations: GU, Genitourinary; N0, Regional nodes not clinically involved; N1, Regional nodes clinically involved by neoplasm; NOS, Not otherwise specified; RMS, rhabdomyosarcoma; VAC, Vincristine/antino mycin/cyclophosphamide; VAC/VI, Vincristine/antino mycin/cyclophosphamide alternating with vincristine/irinotecan.

^a^ Data missing for one patient.

^b^ ERMS/Spindle cell/BRMS were treated as FOXO1−

### 3.2 Relationship between complete metabolic response and EFS

**FIGURE 2:**
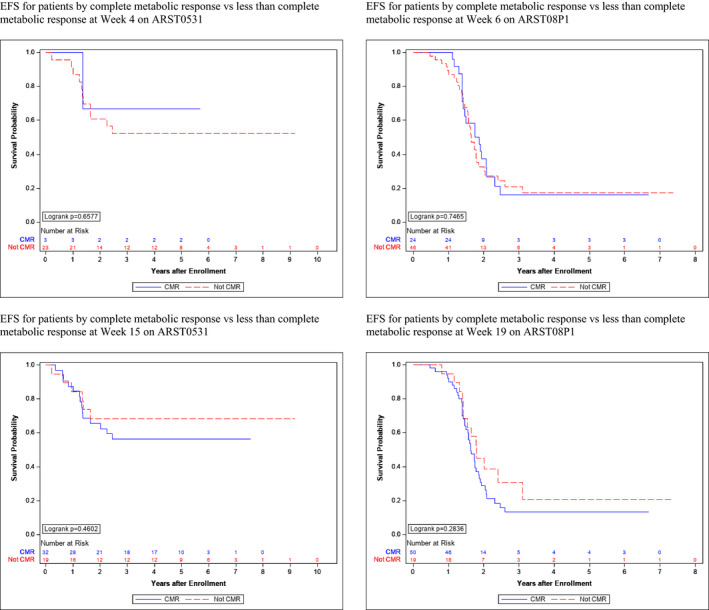
EFS by CMR vs less than CMR for IR and HR RMS Patients

Of the 26 IR RMS patients who had FDG‐PET imaging available for review at week 4 following one cycle of chemotherapy, a CMR was achieved in three (11.5%). No significant improvement in 3‐year EFS was seen in patients who achieved a CMR compared to those who did not at week 4 (3‐year EFS 67% vs 52%, *p* = 0.66). Of the 51 patients who had FDG‐PET imaging available for review at week 15 following five cycles of chemotherapy and local radiotherapy, CMR was achieved in 32 (62.7%). No significant improvement in 3‐year EFS was seen in patients who achieved a CMR compared to those who did not at week 15 (3‐year EFS: 56% vs 68%, *p* = 0.46; Table 2; Figure 2).

**TABLE 2:** Relationship between the presence/absence of complete metabolic response (CMR) and EFS in IR RMS (ARST0531) and HR RMS (ARST08P1) at different timepoints as assessed by central imaging review

PET imaging criterion	N	3 year event‐free survival (95% CI)	*p*‐value
ARST0531			
Response at week 4			
CMR	3	67% (13.3–100%)	0.66
<CMR	23	52% (31.8–72.6%)	
Response at week 15			
CMR	32	56% (39.1–73.4%)	0.46
<CMR	19	68% (46.7–90.2%)	
ARST08P1			
Response at week 6			
CMR	24	16% (0–32.7%)	0.75
<CMR	46	21% (6.0–35.7%)	
Response at week 19			
CMR	50	13% (2.4–24.0%)	0.28
<CMR	19	31% (1.8–59.9%)	

**FIGURE 3:**
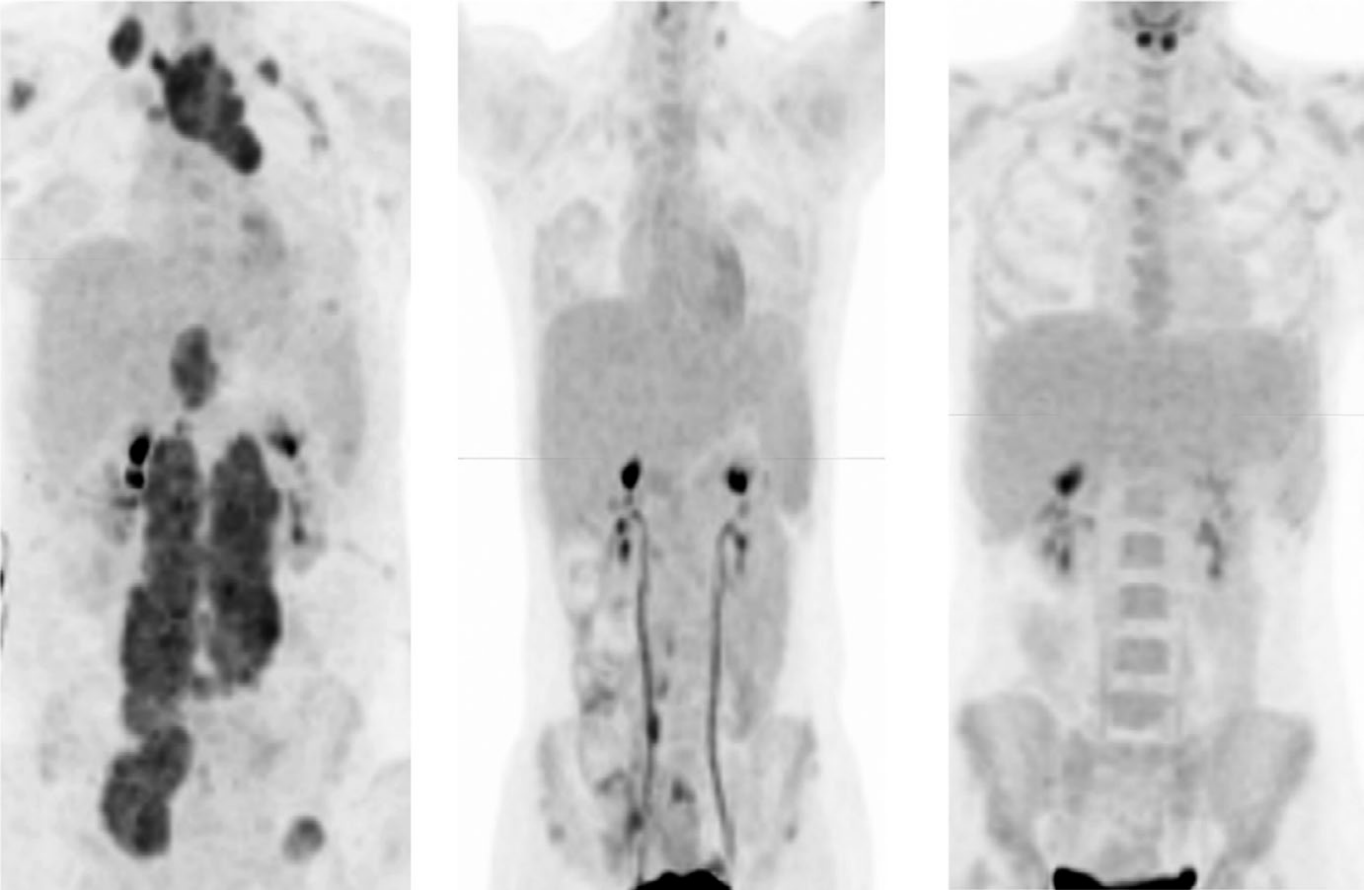
17 year old girl with alveolar RMS of the lower extremity enrolled on ARST08P1. Images at the time of presentation (left panel) show right sided supraclavicular, left anterior mediastinal, subdiaphragmatic, widespread bilateral retroperitoneal, and left iliac bone uptake. By 6 weeks most sites of uptake had resolved. SUVmax of the anterior mediastinal mass declined from 8.7 to 1.7. At 19 weeks no residual areas of abnormal uptake were present. FDG‐PET response does not correlate with EFS in either IR or HR RMS. This patient suffered distant recurrence 6 months following completion of protocol directed therapy despite excellent FDG‐PET response, and eventually died from recurrent disease

Of the 70 HR RMS patients who had FDG‐PET imaging available for review at week 6, a CMR was achieved in 24 (34.3%). No significant improvement in 3‐year EFS was seen in patients who achieved a CMR compared to those who did not at week 6 (3‐year EFS 16% vs 21%, *p* = 0.75). Of the 69 patients who had FDG‐PET imaging available for review at week 19, CMR was achieved in 50 (72.5%). No significant improvement in 3‐year EFS was seen in patients who achieved a CMR compared to those who did not at week 19 (3‐year EFS 13% vs 31%, *p* = 0.28; Table 2; Figures 2 and 3).

### 3.3 Relationship between SUVmax and EFS

The median (range) SUVmax of the primary tumor at diagnosis of IR and HR patients was 6.0 (range, 1.3–24.5) and 7.9 (range, 1.6–26.8), respectively. Increased SUVmax at study entry did not correlate with EFS in IR RMS patients (Hazard ratio: 0.97, *p* = 0.32) or HR RMS (Hazard ratio: 1.00, *p* = 0.86; Table 3).

**TABLE 3:** Relationship between FDG PET maximum standard uptake value (SUVmax) at study entry and ratio of later SUVmax values relative to baseline on EFS as assessed by the Cox proportional hazards model

	Sample size	Hazard ratio	95% CI, for HR	*p*‐value
ARST0531				
SUVmax0	111	0.97	(0.911, 1.031)	0.32
SUVmax4/SUVmax0	32	2.72	(0.569, 13.047)	0.21
SUVmax15/SUVmax0	68	0.95	(0.409, 2.226)	0.91
ARST08P1				
SUVmax0	95	1.00	(0.945, 1.048)	0.86
SUVmax6/SUVmax0	64	1.23	(0.344, 4.420)	0.75
SUVmax19/SUVmax0	50	0.66	(0.128, 3.363)	0.61

To determine if change in SUVmax during therapy was associated with EFS, a ratio of SUVmax during therapy to SUVmax at study entry (SUVmax0) was calculated. For IR RMS there was no difference in EFS based on change in SUVmax at week 4 or week 15. (Hazard ratio SUVmax4/SUVmax0: 2.72, *p* = 0.21; Hazard ratio SUVmax15/SUVmax0: 0.95, *p* = 0.91). Similarly, for HR RMS, there was no difference in EFS based on change in SUVmax at week 6 or week 19. (Hazard ratio SUVmax6/SUVmax0: 1.23, *p* = 0.75; Hazard ratio SUVmax19/SUVmax0: 0.66, *p* = 0.61; Table 3).

## 4 DISCUSSION

This is the first prospective cooperative group study evaluating whether FDG‐PET imaging predicts outcome in RMS patients. We evaluated SUVmax at diagnosis, change in SUVmax with therapy, and CMR during treatment (both early CMR following 4 and 6 weeks of chemotherapy in IR RMS and HR RMS, respectively, and late CMR following 15 and 19 weeks of chemotherapy in IR RMS and HR RMS, respectively); none of these parameters predicted EFS in either IR or HR RMS. We conclude that, at the timepoints we used, FDG‐PET is not a useful tool to predict outcome for either IR or HR RMS.

Metabolic assessment of tumors using FDG‐PET evaluation remains a relatively novel modality for assessing response and how best to incorporate this imaging modality into treatment paradigms is an active area of research. Multiple studies have documented FDG‐PET response to be a predictor of outcome in several malignancies including Hodgkin lymphoma,^28^ renal cell carcinoma,^29^ non‐small cell lung cancer,^30^ Ewing sarcoma,^31^ and osteosarcoma.^32^, ^33^, ^34^ A potential prognostic relevance for metabolic response as assessed by FDG‐PET in pediatric RMS is supported by the adult STS literature.^35^, ^36^ Adult patients with high‐grade STS who attain an early metabolic response as defined by a greater than 26% drop in SUVmax after one cycle of chemotherapy have a significantly improved overall survival (OS). Late metabolic response following chemotherapy significantly correlates with OS in univariate but not multivariate analysis.^36^ Moreover, several studies in the STS patient population have shown SUVmax at diagnosis to be a significant predictor of outcome, although these have been limited by small sample size and the inclusion of several differing histologic subtypes and tumor primary sites.^36^, ^37^, ^38^

Since RMS is uncommon in adults, it has rarely been represented in STS studies conducted evaluating the utility of FDG‐PET.^39^ The value of FDG‐PET imaging for patients with RMS, thus, remains an open question. In osteosarcoma, the most common primary malignant sarcoma of bone in both the pediatric and adult population, FDG‐PET has been shown to be a potential predictor of histologic response following neoadjuvant chemotherapy, a well‐documented surrogate for outcome in this disease.^31^, ^32^, ^33^, ^40^, ^41^, ^42^ While our results do not support the use of FDG‐PET to predict outcome in IR or HR RMS, several studies have documented the value of FDG‐PET imaging for disease staging. For example, FDG‐PET has been found to have increased sensitivity and specificity compared to conventional imaging including ^99m^Technetium methylene diphosphanate bone scintigraphy in the identification of distant RMS metastases, particularly bone and distant nodal metastases.^20^, ^21^ A retrospective evaluation of FDG‐PET showed improved sensitivity and specificity in identifying nodal metastases in RMS when compared with conventional imaging.^43^ This finding was confirmed in a recent prospective study.^44^ This study, however, documented a low concordance rate between FDG‐PET and pathology after tissue biopsy with nodal tissue sampling and thus, tissue sampling should remain the gold standard when defining sites requiring local control with radiotherapy.^44^ At present, the data clearly support a role for FDG‐PET imaging in the initial staging evaluation of RMS.

Our data contrast with prior single institution reports that supported a role for using CMR to predict EFS in patients with Group III RMS following 15 weeks of chemotherapy and radiation therapy for local control.^22^, ^23^ The reasons for this discrepancy remain unclear. The prior studies were performed at a single institution where the timing of the FDG‐PET imaging and techniques used could be well controlled, as opposed to our cooperative group study which relied on imaging performed at multiple institutions. Furthermore, the ARST0531 study requested FDG‐PET imaging at Week 4 immediately prior to radiotherapy and at Week 15, approximately 6 weeks after completion of radiotherapy. Both of these timepoints are significantly earlier than the above single institution studies that delivered radiation therapy at week 15.

This study has several limitations, most notably the small sample size of the patient population available for this analysis—particularly the small number of paired baseline and follow‐up studies at each timepoint. FDG‐PET imaging was not a requirement for enrollment on either study, and there may have been bias introduced by selective submission of imaging studies, although we could detect no significant differences in the clinical features of the population with and without FDG‐PET imaging available for review (data not shown). Several limitations similarly exist regarding the collection of the FDG‐PET data. While the guidelines for FDG‐PET imaging were specific in both protocols, precise standardization of imaging equipment was not practical due to the large number of institutions involved.^45^ Furthermore, while several FDG‐PET imaging variables and their relationship to outcome were evaluated, more advanced parameters such as metabolic tumor volume and total lesion glycolysis that were beyond the scope of this analysis were not evaluated although could be considered for future analyses.^46^ Finally, FDG‐PET imaging was performed at diagnosis, week 4 and week 15 of therapy in ARST0531, and at diagnosis, week 6, and week 19 in ARST08P1. It remains possible that FDG‐PET response later in therapy, immediately following local control, or at the conclusion of all planned treatment could potentially be prognostic.

In conclusion, our analysis does not support using FDG‐PET as a predictor for outcome in the IR or HR RMS population. Further prospective studies are needed to determine whether this imaging modality has a role in predicting response to therapy or outcome in other RMS subpopulations, and whether FDG‐PET imaging at a later timepoint would be more predictive of outcome. Additional research is needed to identify other methods to measure therapy response that can reliably predict outcome in RMS.

## 5 ETHICAL APPROVAL STATEMENT

The planning, conduct, and reporting of this research are in accordance with the Helsinki Declaration as revised in 2013 (www.wma.net/policies‐post/wma‐declaration‐of‐helsinkiethical‐principles‐for‐medical‐research‐involving‐humansubjects/). The research was approved by institutional review board as per ICJME guidelines.

## CONFLICT OF INTEREST

The authors have no conflict of interest.

## AUTHOR CONTRIBUTIONS

Each author has made substantial contributions to the conception and design, acquisition of data, and analysis and interpretation of data of this project. Each has been involved in drafting the manuscript or revising it critically for important intellectual content. All have reviewed the final manuscript and agreed upon the manuscript content.

## Data Availability

Children's Oncology Group Data Sharing Statement: The Children's Oncology Group Data Sharing policy describes the release and use of COG individual subject data for use in research projects in accordance with National Clinical Trials Network (NCTN) Program and NCI Community Oncology Research Program (NCORP) Guidelines. Only data expressly released from the oversight of the relevant COG Data and Safety Monitoring Committee (DSMC) are available to be shared. Data sharing will ordinarily be considered only after the primary study manuscript is accepted for publication. For phase 3 studies, individual‐level de‐identified datasets that would be sufficient to reproduce results provided in a publication containing the primary study analysis can be requested from the NCTN/NCORP Data Archive at https://nctn‐data‐archive.nci.nih.gov/. Data are available to researchers who wish to analyze the data in secondary studies to enhance the public health benefit of the original work and agree to the terms and conditions of use. For non‐phase 3 studies, data are available following the primary publication. An individual‐level de‐identified dataset containing the variables analyzed in the primary results paper can be expected to be available upon request. Requests for access to COG protocol research data should be sent to: datarequest@childrensoncologygroup.org. Data are available to researchers whose proposed analysis is found by COG to be feasible and of scientific merit and who agree to the terms and conditions of use. For all requests, no other study documents, including the protocol, will be made available and no end date exists for requests. In addition to above, release of data collected in a clinical trial conducted under a binding collaborative agreement between COG or the NCI Cancer Therapy Evaluation Program (CTEP) and a pharmaceutical/biotechnology company must comply with the data sharing terms of the binding collaborative/contractual agreement and must receive the proper approvals.

## References

[1] Gurney JG , Young JL , Roffers SD , et al. Soft tissue sarcomas In Ries LAG , Smith MA , Gurney JG (eds). Cancer Incidence and Survival among Children and Adolescents: United States SEER Program 1975–1995. Bethesda, MD, 19.

[2] Hawkins DS , Spunt SL , Skapek SX . Children's Oncology Group's 2013 blueprint for research: soft tissue sarcomas. Pediatr Blood Cancer. 2013;60:1001‐1008. 23255356 10.1002/pbc.24435 PMC3777409

[3] Skapek SX , Ferrari A , Gupta A , et al. Rhabdomyosarcoma. Nat Rev Dis Primers. 2019;5(1):1‐19. 30617281 10.1038/s41572-018-0051-2 PMC7456566

[4] Walterhouse DO , Pappo AS , Meza JL , et al. Shorter‐duration therapy using vincristine, dactinomycin, and lower‐dose cyclophosphamide with or without radiotherapy for patients with newly diagnosed low‐risk rhabdomyosarcoma: a report from the Soft Tissue Sarcoma Committee of the Children’s Oncology Group. J Clin Oncol. 2014;32(31):3547‐3552. 25267746 10.1200/JCO.2014.55.6787 PMC4209105

[5] Hawkins DS , Chi YY , Anderson JR , et al. Addition of vincristine and irinotecan to vincristine, dactinomycin, and cyclophosphamide does not improve outcome for intermediate‐risk rhabdomyosarcoma: a report from the Children's Oncology Group. J Clin Oncol. 2018;36(27):2770‐2777. 30091945 10.1200/JCO.2018.77.9694 PMC6145831

[6] Weigel BJ , Lyden E , Anderson JR , et al. Intensive multiagent therapy, including dose‐compressed cycles of ifosfamide/etoposide and vincristine/doxorubicin/cyclophosphamide, irinotecan, and radiation, in patients with high‐risk Rhabdomyosarcoma: A report from the Children's Oncology Group. J Clin Oncol. 2016;34(2):117‐122. 26503200 10.1200/JCO.2015.63.4048 PMC5070550

[7] Malempati S , Weigel BJ , Chi YY , et al. The addition of cixutumumab or temozolomide to intensive multiagent chemotherapy is feasible but does not improve outcome for patients with metastatic rhabdomyosarcoma: a report from the Children’s Oncology Group. Cancer. 2019;125(2):290‐297. 30351457 10.1002/cncr.31770 PMC6329653

[8] Oberlin O , Rey A , Lyden E , et al. Prognostic factors in metastatic rhabdomyosarcomas: results of a pooled analysis from United States and European Cooperative Groups. J Clin Oncol. 2008;26(14):2384‐2389. 18467730 10.1200/JCO.2007.14.7207 PMC4558625

[9] Breneman JC , Lyden E , Pappo AS , et al. Prognostic factors and clinical outcomes in children and adolescents with metastatic rhabdomyosarcoma‐a report from the Intergroup Rhabdomyosarcoma Study IV. J Clin Oncol. 2003;21(1):78‐84. 12506174 10.1200/JCO.2003.06.129

[10] Chisholm JC , Merks JHM , Casanova M , et al. Open‐label, multicentre, randomised, phase II study of the EpSSG and the ITCC evaluating the addition of bevacizumab to chemotherapy in childhood and adolescent patients with metastatic soft tissue sarcoma (the BERNIE study). Eur J Cancer. 2017;83:177‐184. 28738258 10.1016/j.ejca.2017.06.015

[11] Visser JH , Wessels G , Hesseling PB , et al. Prognostic value of day 14 blast percentage and the absolute blast index in bone marrow of children with acute lymphoblastic leukemia. Pediatr Hematol Oncol. 2001;18:187‐191. 11293286 10.1080/08880010151114804

[12] Gaynon PS , Bleye QA , Steinherz PG , et al. Day 7 marrow response and outcome for children with acute lymphoblastic leukemia and unfavorable presenting features. Med Pediatr Oncol. 1990;18:273‐279. 2355886 10.1002/mpo.2950180403

[13] Weiner MA , Leventhal B , Brecher ML , et al. Randomized study of intensive MOPP‐ABVD with or without low‐dose total‐nodal radiation therapy in the treatment of stages IIB, IIIA2, IIIB, and IV Hodgkin’s disease in pediatric patients: A Pediatric Oncology Group study. J Clin Oncol. 1997;15:2769‐2779. 9256118 10.1200/JCO.1997.15.8.2769

[14] Picci P , Rougraff BT , Bacci G , et al. Prognostic significance of histopathologic response to chemotherapy in nonmetastatic Ewing’s sarcoma of the extremities. J Clin Oncol. 1993;11:1763‐1769. 8355043 10.1200/JCO.1993.11.9.1763

[15] Wunder JS , Paulian G , Huvos AG , et al. The histological response to chemotherapy as a predictor of the oncological outcome of operative treatment of Ewing sarcoma. J Bone Joint Surg Am. 1998;80:1020‐1033. 9698007 10.2106/00004623-199807000-00011

[16] Rosen G , Caparros B , Groshen S , et al. Primary osteogenic sarcoma of the femur: a model for the use of preoperative chemotherapy in high risk bone tumors. Cancer Invest. 1984;2:181‐192. 6203625 10.3109/07357908409104370

[17] Burke M , Anderson JR , Kao SC , et al. Assessment of response to induction therapy and its influence on 5‐year failure‐free survival in group III rhabdomyosarcoma: the Intergroup Rhabdomyosarcoma Study‐IV experience–a report from the Soft Tissue Sarcoma Committee of the Children's Oncology Group. J Clin Oncol. 2007;25(31):4909‐4913. 17971587 10.1200/JCO.2006.10.4257

[18] Rosenberg AR , Anderson JR , Lyden E , et al. Early response as assessed by anatomic imaging does not predict failure‐free survival among patients with Group III rhabdomyosarcoma: a report from the Children’s Oncology Group. Eur J Cancer. 2014;50(4):816‐823. 24361229 10.1016/j.ejca.2013.11.031 PMC3944684

[19] Vaarwerk B , van der Lee JH , Breunis WB , et al. Prognostic relevance of early radiologic response to induction chemotherapy in pediatric rhabdomyosarcoma: a report from the International Society of Pediatric Oncology Malignant Mesenchymal Tumor 95 study. Cancer. 2018;124(5):1016‐1024. 29211298 10.1002/cncr.31157

[20] Federico SM , Spunt SL , Krasin MJ , et al. Comparison of PET‐CT and conventional imaging in staging pediatric rhabdomyosarcoma. Pediatr Blood Cancer. 2013;60(7):1128‐1134. 23255260 10.1002/pbc.24430 PMC4266929

[21] Eugene T , Corradini N , Carlier T , et al. ^1^⁸F‐FDG‐PET/CT in initial staging and assessment of early response to chemotherapy of pediatric rhabdomyosarcomas. Nucl Med Commun. 2012;33(10):1089‐1095. 22929116 10.1097/MNM.0b013e328356741f

[22] Dharmarajan KV , Wexler LH , Tom A , et al. Positron emission tomography (PET) response to initial chemotherapy and radiation therapy predicts local control in rhabdomyosarcoma. Int J Radiat Oncol Biol Phys. 2012;84(4):996‐1002. 22560547 10.1016/j.ijrobp.2012.01.077

[23] Casey DL , Wexler LH , Fox JJ , et al. Predicting outcome in patients with rhabdomyosarcoma: role of [^18^F] fluorodeoxyglucose positron emission tomography. Int J Radiat Oncol Biol Phys. 2014;90(5):1136‐1142. 25539372 10.1016/j.ijrobp.2014.08.005

[24] Young H , Baum R , Cremerius U , et al. Measurement of clinical and subclinical tumour response using [18F]‐fluorodeoxyglucose and positron emission tomography: Review and 1999 EORTC recommendations. European Organization for Research and Treatment of Cancer (EORTC) PET study group. Eur J Cancer. 1999;35(13):1773‐1782. 10673991 10.1016/s0959-8049(99)00229-4

[25] Kaplan GL , Meier P . Nonparametric estimation from incomplete observations. J Am Stats Assoc. 1958;53:457‐481.

[26] Peto R , Pike MC , Armitage P ,. et al. Design and analysis of randomized clinical trials requiring prolonged observations of each patient. II. Analysis and examples. Br J Cancer. 1977;35:1‐39. 831755 10.1038/bjc.1977.1 PMC2025310

[27] Cox DR . Regression models and life‐tables. J R Stat Soc Series B. 1972;34:187‐220.

[28] Friedman DL , Chen L , Wolden S , et al. Dose‐Intensive response based chemotherapy and radiation therapy for children and adolescents with newly diagnosed intermediate‐risk Hodgkin lymphoma: a report from the Children’s Oncology Group Study AHOD0031. J Clin Oncol. 2014;32(32):3651‐3658. 25311218 10.1200/JCO.2013.52.5410 PMC4220044

[29] Farnebo J , Grybäck P , Harmenberg U , et al. Volumetric FDG‐PET predicts overall and progression‐free survival after 14 days of targeted therapy in metastatic renal cell carcinoma. BMC Cancer. 2014;14(408). 10.1186/1471-2407-14-408. PMC4064288 24906441

[30] Ding Q , Cheng X , Yang L , et al. PET/CT evaluation of response to chemotherapy in non‐small cell lung cancer: PET response criteria in solid tumors (PERCIST) versus response evaluation criteria in solid tumors (RECIST). J Thorac Dis. 2014;6(6):677‐683. 24976990 10.3978/j.issn.2072-1439.2014.05.10 PMC4073366

[31] Hawkins DS , Schuetze SM , Butrynski JE , et al. [^18^F]‐fluorodeoxy‐D‐glucose positron emission tomography predicts outcome for Ewing sarcoma family of tumors. J Clin Onco. 2005;23:8828‐8834. 10.1200/JCO.2005.01.7079 16314643

[32] Hawkins DS , Conrad EU , Butrynski JE , Schuetze SM , Eary JF . [F‐18]‐fluorodeoxy‐D‐glucose positron emission tomography response is associated with outcome for extremity osteosarcoma in children and young adults. Cancer. 2009;115:3519‐3525. 19517457 10.1002/cncr.24421 PMC2716419

[33] Davis JC , Daw NC , Navid F , et al. ^18^F‐FDG uptake during early adjuvant chemotherapy predicts histologic response in pediatric and young adult patients with osteosarcoma. J Nucl Med. 2018;59:25‐30. 28611244 10.2967/jnumed.117.190595 PMC5750521

[34] Costelloe CM , Macapiniac HA , Madewell JE , et al. 18F‐FDG PET/CT as an indicator of progression‐free and overall survival in osteosarcoma. J Nucl Med. 2009;50(3):340‐347. 19258257 10.2967/jnumed.108.058461

[35] Hong S , Lee SE , Choi Y , et al. Prognostic value of^18^F‐FDG PET/CT in patients with soft tissue sarcoma: comparisons between metabolic parameters. Skeletal Radiol. 2014;43:641‐648. 24531303 10.1007/s00256-014-1832-7

[36] Herrmann K , Benz MR , Czernin J , et al. 18F‐FDG‐PET/CT imaging as an early survival predictor in patients with primary high‐grade soft tissue sarcomas undergoing neoadjuvant therapy. Clin Cancer Res. 2012;18(7):2024‐2031. 22338012 10.1158/1078-0432.CCR-11-2139 PMC3431618

[37] Eary JF , Conrad EU , O'Sullivan J , et al. Sarcoma mid‐therapy [F‐18] fluorodeoxyglucose positron emission tomography (FDG PET) and patient outcome. J Bone Joint Surg Am. 2014;96:152‐158. 24430415 10.2106/JBJS.M.00062 PMC3903137

[38] Skamene SR , Rakheja R , Dahlstrom KR , et al. Metabolic activity measured on PET/CT correlates with clinical outcomes in patients with limb and girdle sarcomas. J Surg Oncol. 2014;109(5):410‐414. 24310279 10.1002/jso.23523

[39] Gennaro N , Marrari RSL , et al. Multimodality imaging of adult rhabdomyosarcoma: the added value of hybrid imaging. Br J Radiol. 2020;93(1112):20200250. 32559113 10.1259/bjr.20200250 PMC7446015

[40] Byun BH , Kong CB , Park J , et al. Comparison of (18)F‐FDG PET/CT and (99 m)Tc‐MDP bone scintigraphy for detection of bone metastasis in osteosarcoma. Skeletal Radiol. 2013;42(12):1673‐1681. 23995264 10.1007/s00256-013-1714-4

[41] Byun BH , Kong C , Lim I , et al. Early response monitoring to neoadjuvant chemotherapy in osteosarcoma using sequential^18^F‐FDG PET/CT and MRI. Eur J Nucl Med Mol Imaging. 2014;41:1553‐1562. 24652233 10.1007/s00259-014-2746-2

[42] Byun BH , Kong C , Lim I , et al. Combination of ^18^F‐FDG PET/CT and diffusion weighted MR imaging as a predictor of histologic response to neoadjuvant chemotherapy: preliminary results in osteosarcoma. J Nucl Med. 2013;54(7):1053‐1059. 23670899 10.2967/jnumed.112.115964

[43] Norman G , Fayter D , Lewis‐Light K , et al. An emerging evidence base for PET‐CT in the management of childhood rhabdomyosarcoma: a systematic review. BMJ Open. 2015;5(1):1‐8. 10.1136/bmjopen-2014-006030 PMC4289735 25573522

[44] Wagner LM , Kremer N , Gelfand MJ , et al. Detection of lymph node metastases in pediatric and adolescent/young adult sarcoma: sentinel lymph node biopsy versus fludeoxyglucose positron emission tomography imaging – a prospective trial. Cancer. 2016;123(1):155‐160. 27563842 10.1002/cncr.30282

[45] Scheuermann JS1 , Saffer JR , Karp JS , et al. Qualification of PET scanners for use in multicenter cancer clinical trials: the American College of Radiology Imaging Network experience. J Nucl Med. 2009;50(7):1187‐1193. 19525463 10.2967/jnumed.108.057455 PMC2744888

[46] Im HJ , Zhang Y , Wu H , et al. Prognostic value of metabolic and volumetric parameters of FDG PET in pediatric osteosarcoma: a hypothesis‐generating study. Radiology. 2018;287(1):303‐312. 29357275 10.1148/radiol.2017162758

